# Comprehensive characterization of tumor microenvironment and m6A RNA methylation regulators and its effects on PD-L1 and immune infiltrates in cervical cancer

**DOI:** 10.3389/fimmu.2022.976107

**Published:** 2022-08-26

**Authors:** Huihui Ji, Jian-an Zhang, Hejing Liu, Kehan Li, Zhi-wei Wang, Xueqiong Zhu

**Affiliations:** Center of Uterine Cancer Diagnosis and Therapy Research of Zhejiang Province, Department of Obstetrics and Gynecology, The Second Affiliated Hospital of Wenzhou Medical University, Wenzhou, China

**Keywords:** cervical cancer, M6A, TME, PD-1, PD-L1, TIME

## Abstract

Understanding the role of N6-adenosine methylation (m6A) in the tumor microenvironment (TME) is important since it can contribute to tumor development. However, the research investigating the association between m6A and TME and cervical cancer is still in its early stages. The aim of this study was to discover the possible relationship between m6A RNA methylation regulators, TME, PD-L1 expression levels, and immune infiltration in cervical cancer. We gathered RNA-seq transcriptome data and clinical information from cervical cancer patients using The Cancer Genome Atlas (TCGA) and Genotype-Tissue Expression (GTEx) databases. To begin, researchers assessed the differences in m6A regulatory factor expression levels between cervical cancer and normal tissues. Clustering analysis was adapted to assess PD-L1 expression, immunological score, immune cell infiltration, TME, and probable pathways in cervical cancer samples. The majority of m6A regulators were found to be considerably overexpressed in cervical cancer tissues. Using consensus clustering of 21 m6A regulators, we identified two subtypes (clusters 1/2) of cervical cancer, and we found that WHO stage and grade were associated with the subtypes. PD-L1 expression increased dramatically in cervical cancer tissues and was significantly linked to ALKBH5, FTO, METTL3, RBM15B, YTHDF1, YTHDF3, and ZC3H13 expression levels. Plasma cells and regulatory T cells (Tregs) were considerably elevated in cluster 2. Cluster 1 is involved in numerous signature pathways, including basal transcription factors, cell cycle, RNA degradation, and the spliceosome. The prognostic signature-based riskscore (METTL16, YTHDF1, and ZC3H13) was found to be an independent prognostic indicator of cervical cancer. The tumor immune microenvironment (TIME) was linked to m6A methylation regulators, and changes in their copy number will affect the quantity of tumor-infiltrating immune cells dynamically. Overall, our research discovered a powerful predictive signature based on m6A RNA methylation regulators. This signature correctly predicted the prognosis of cervical cancer patients. The m6A methylation regulator could be a critical mediator of PD-L1 expression and immune cell infiltration, and it could have a significant impact on the TIME of cervical cancer.

## Introduction

Cervical cancer is the fourth most common cancer among women in the world, and it has 604,000 new cases and 342,000 deaths in 2020 (1). Long-term infection with human papillomavirus (HPV) may be a main risk factor for cervical cancer development (2, 3). The major treatment strategies for cervical cancer are hysterectomy, radiotherapy, chemotherapy, and radiochemotherapy (4). However, recurrent and metastatic cervical cancer has a dismal prognosis (5). Recently, immunotherapy has advanced fast, extending the survival in advanced and metastatic cancer patients who are previously thought to be incurable (6). However, only a small percentage of cervical cancer patients may benefit from immunotherapy due to individual differences (7).

Immune system dysregulation plays a key role in the development of cervical cancer. For instance, the density of CD4+ and CD8+ T-cell infiltrate correlated with the severity of lesions in cervical cancer (8). When compared to stage I cervical cancer patients, researchers discovered that stage II patients had lower levels of circulating Th1 cells and higher levels of Th2 cells, Th17 cells, and regulatory T cells (Tregs) (9). Moreover, accumulated evidence suggested that increased Treg levels were also found at the cervical tumor site and in the lymph nodes of cervical cancer patients (10, 11). It is believed that the mechanism of the tumor immune microenvironment (TIME) needs to be further investigated in order to have a better treatment effect on cervical cancer patients.

Post-transcriptional modification (PTM) is thought to be involved in the progression of many diseases (12). N6-adenosine methylation (m6A) is the most abundant RNA modification, and plays crucial roles in multiple physiological processes and disease progression (13, 14). Currently, m6A has a variety of methyltransferases, including m6A “writers” (METTL3, METTL14, METTL16, WTAP, KIAA1429, RBM15, RBM15B, and ZC3H13), m6A “erasers” (FTO and ALKBH5) and m6A “readers” (EIF3A, IGF2BP1, IGF2BP2, IGF2BP3, YTHDC1, YTHDC2, YTHDF1, YTHDF2, YTHDF3, HNRNPC, and HNRNPA2B1) (15). Accumulated evidence has proved that m6A is correlated with the progression and prognosis of cervical cancer (13). For instance, patients with greater levels of YTHDF1 in cervical cancer had a poor prognosis (13), and YTHDF1 knockdown reduced the carcinogenesis of cervical cancer cells (16). Another study found that METTL3 was highly increased in cervical cancer tissue and cells, which was linked to lymph node metastases and a poor prognosis in cervical cancer patients (17). METTL3 accelerated cervical oncogenesis and Warburg effect *via* modification of YTHDF1/HK2 (17). However, the mechanisms of other m6A methylation regulators in cervical cancer remain unclear. Moreover, the association between m6A methylation regulators and programmed death ligand 1 (PD-L1) need to be further studied.

**Figure 1:**
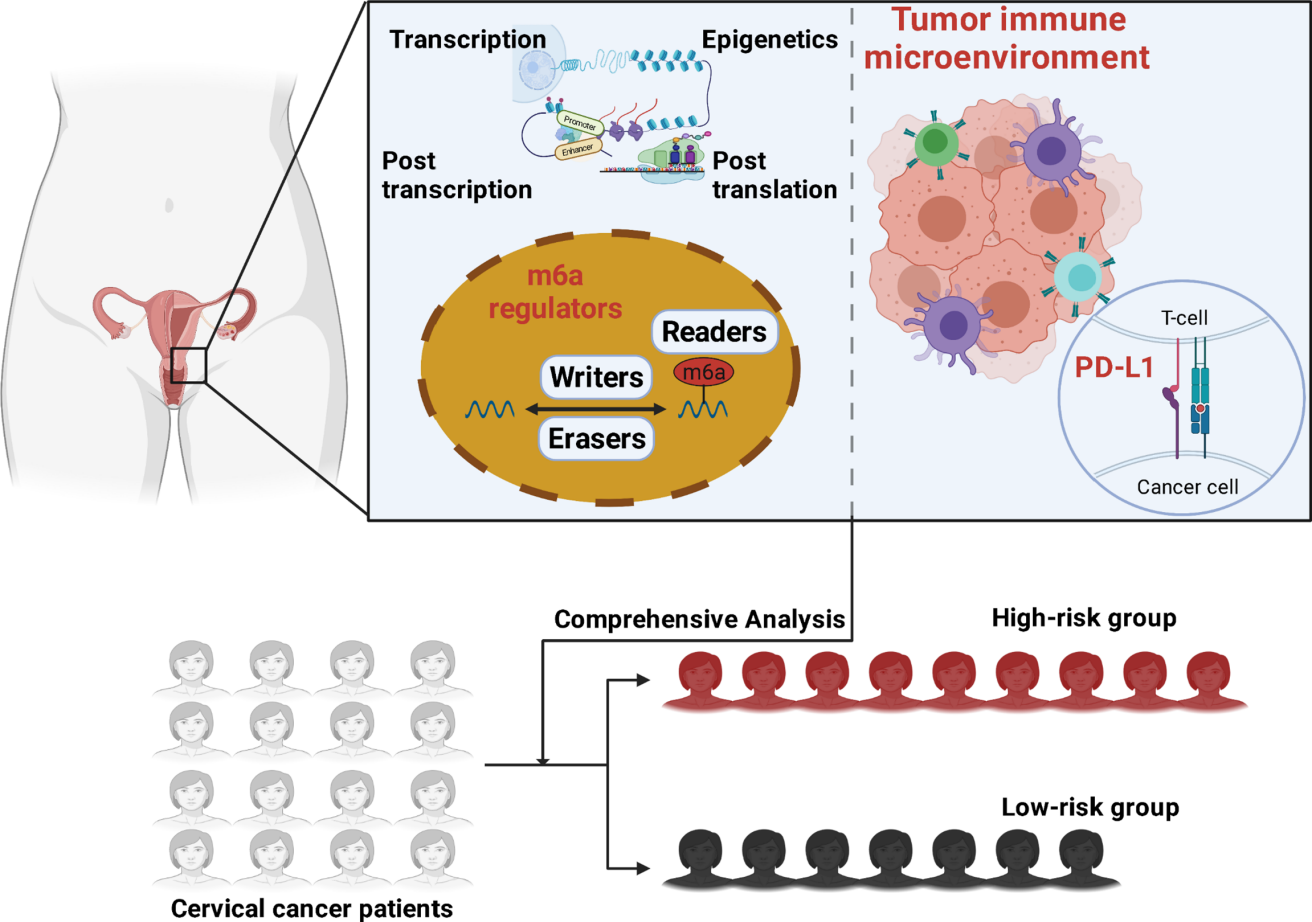
Graphical abstract is illustrated.

Our research investigated the relationship between m6A methylation regulators and PD-L1, prognosis, and TIME in cervical cancer. Furthermore, we separated the TCGA cohort into two clusters and developed a signature based on m6A methylation regulators to better predictive risk classification and treatment decisions in patients with cervical cancer. We thoroughly investigated the association between clustering subgroups, risk models, PD-L1 expression, immunescores, and immune cell infiltration. The study also aimed to explore potential regulatory pathways regulating TIME and cervical cancer therapeutic methods (**Figure 1**).

## Materials and methods

### Dataset acquisition

This study gained data from The Cancer Genome Atlas (TCGA-CESC) cohort. The mRNA expression data and corresponding clinicopathological data were obtained from 304 cervical cancer patients. Meanwhile, mRNA expression data were from three cervical cancer adjacent tissues. The Genotype-Tissue Expression (GTEx) data gateway was also used to gather mRNA expression data from 10 normal cervical epithelial tissues. Since the TCGA-CESC clinical data contain all cervical cancer patients, there are varying numbers of cervical cancer patients for mRNA expression patterns, which we will match later. The mRNA expression data were normalized by fragment per kilobase of exon model per million (FPKM, mean fragment per kilobase million). Clinicopathological information included survival state, survival time, age, HPV status, cisplatin using, grade, and Tumor Node Metastasis (TNM) staging.

### Detection of 21 m6A regulators

In this study, 21 genes (METTL3, METTL14, METTL16, WTAP, KIAA1429, RBM15, RBM15B, ZC3H13, FTO, ALKBH5, EIF3A, IGF2BP1, IGF2BP2, IGF2BP3, YTHDC1, YTHDC2, YTHDF1, YTHDF2, YTHDF3, HNRNPC, and HNRNPA2B1) were selected as classical m6A regulators. The mRNA expression data were then used to extract the expression of the 21 m6A regulators. The “pheatmap” and “vioplot” R packages were used to create a heatmap and a violin plot to better visualize the difference in m6A methylation regulators between cervical cancer and control groups. Additionally, mutations in those genes were retrieved from mutation annotation format (MAF) data and shown as a waterfall plot using the oncostrip function in the “maftools” package. Moreover, many studies found that tumor mutation burden (TMB) and neoepitopes were strongly linked to immunotherapy in a variety of cancer types. Therefore, the TMB of each sample was also calculated by the “maftools” R package. We used The Search Tool for the Retrieval of Interacting Genes/Proteins database to better understand m6A interactions (STRING, version 11.0, http://string-db.org/) (18). Then, this study employed Gene Ontology (GO) to analyze a preliminary understanding of their biological functions of 21 m6A methylation regulators. Furthermore, the correlation between different m6A methylation regulators was elucidated using Spearman correlation analysis.

### Prognostic signature model

We screened the signature in 21 m6A regulators using univariate Cox regression, with a hazard ratio of more than 1 indicating a higher risk and a hazard ratio of less than 1 indicating a lower risk. Then, using the least absolute shrinkage and selection operator (LASSO) regression, we created predictive risk signatures for m6A regulators. The coefficients obtained from the LASSO regression were used to yield the following riskscore equation: riskscore = sum of coefficients * m6A regulator expression levels. In the current investigation, this method was used to calculate the riskscore of each cancer cervical patient. The median riskscore was utilized as the cutoff criterion for categorizing the samples into high-risk and low-risk groups.

### Evaluating the prognostic value of the m6A signature

To analyze the difference in overall survival (OS) between the high- and low-risk groups, a Kaplan–Meier analysis was used. Receiver operating characteristic (ROC) curves were used to examine the prognostic capacity of the riskscores, and the area under the curve (AUC) was calculated. The distribution of clinicopathological traits in high- and low-risk groups was depicted using the R package “heatmap”. Cox regression models were employed in univariate and multivariate analyses to see whether riskscores may serve as independent prognostic indicators when combined with other clinical features.

### PD-L1 genomic variation and co-expression level

The cBioPortal tool was used to identify possible PD-L1 copy number variations and mutations in cervical cancer. OncoPrint displayed a summary of PD-L1 genomic changes in cervical cancer samples. The changes in PD-L1 expression between tumor samples and normal samples, the two clusters, and the high- and low-risk groups were also depicted in this study. The link between PD-L1 expression and m6A regulators was calculated using Spearman correlation.

### The relationship between m6A regulators and TIME in cervical cancer

Each cervical cancer patient’s immunescore was calculated using the R programme “estimate.” The fraction of 22 immune cell subtypes in each cervical cancer sample was determined by determining relative subtypes of RNA transcripts. This study has adapted the 1,000 permutations algorithm clustering, and riskscores were used to compare differences in immune infiltration levels between subgroups. Tumor Immune Estimation Resource assessed the influence of somatic copy number alternations (CNAs) in m6A regulators on the amount of immune cell infiltration.

### Cell culture

Human cervical cancer cells (HeLa and CaSki) were purchased from the National Collection of Authenticated Cell Cultures. Cells were maintained with Dulbecco’s modified Eagle’s medium (DMEM, Gibco, USA) or RPMI 1640 medium supplemented with 10% fetal bovine serum (Gibco, USA) at 37°C with 5% CO_2_.

### Immunohistochemical staining

All tissues were obtained from cervical cancer patients who underwent radical hysterectomy at the Second Affiliated Hospital of Wenzhou Medical University. Patients did not receive chemotherapy or radiotherapy before surgery. IHC was performed in cancer tissues and surrounding normal tissues using a previously described method (19). This study was approved by the ethics committee of the Second Affiliated Hospital of Wenzhou Medical University.

### Western blot analysis

The siRNA sequences were purchased from Tsingke Biotechnology Co. and listed in **Supplementary Table 1**. The transfected HeLa and CaSki cells were treated with radio immunoprecipitation assay (RIPA) lysis buffer. Bicinchoninic acid (BCA) protein determination kits were applied to extract and quantify the total protein in the cells. Proteins were electrophoresed in a PVDF membranes. Membranes were incubated with primary antibodies at 4°C overnight. PD-L1 (1:1,000; ab213524; Abcam), YTHDF1 (1:1,000; ab230330; Abcam), METTL16 (1:300; orb679493; Biorbyt), ZC3H13 (1:1,000; ab70802; Abcam), and GAPDH (1:3,000; AB-M-M 001; GoodHere Technology) were purchased and used in this study. The Western blotting analysis was performed as described before (20).

### Statistical analysis

All statistical tests were performed using R software (version 3.6.1). The Wilcoxon test and one-way analysis of variance (ANOVA) were used to calculate differences between two groups and among several groups. Kaplan–Meier analysis and a log-rank test were used to compare the OS of the two groups. A Spearman correlation test was used to evaluate the subtypes, clinicopathological characteristics, riskscores, PD-L1 expression, and immune infiltration levels. Only a *p*-value below 0.05 was regarded statistically significant.

## Results

### The m6A methylation regulators are expressed differently in cervical cancer

**Figure 2:**
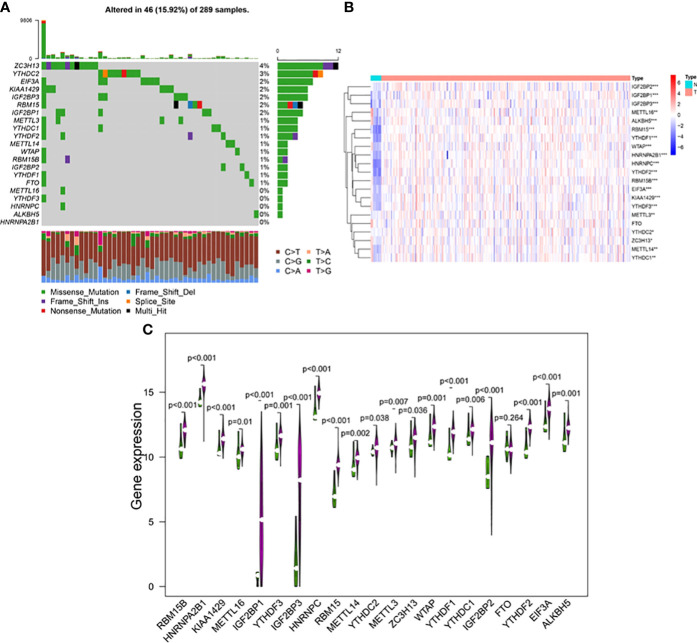
**(A)** The mutation frequency of 21 m6A regulators in 289 patients with cervical cancer from the TCGA-CESC cohort. Each column represented individual patients. The upper bar plot showed TMB, and the number on the right indicated the mutation frequency in each regulator. The right bar plot showed the proportion of each variant type. The stacked bar plot below showed fraction of conversions in each sample. The expression levels of m6A RNA methylation regulators between tumor and normal samples in TCGA CESC and GTEx cohorts. **(B)** Heatmap of m6A RNA methylation regulator expression level in each sample. **(C)** The expression difference of m6A RNA methylation regulator between tumor and normal samples. *p < 0.05; **p < 0.01; ***p < 0.001.

The occurrence of copy number variations and somatic mutations of 21 m6A regulators in cervical cancer was initially summarized. Mutations of m6A regulators were found in 46 of the 289 samples, with a frequency of 15.92% (**Figure 2A**). The highest mutation frequency was found in ZC3H13, followed by YTHDC2. Moreover, WTAP, KIAA1429, EIF3, IGF2BP1, IGF2BP2, IGF2BP3, RBM15, RBM15B, YTHDF1, YTHDF2, YTHDF3, HNRNPC, HNRNPA2B1, and ALKBH5 had a high expression in cervical cancer tumor tissues as compared to 13 normal and 304 malignant tissues. METTL3, METTL14, METTL16, YTHDC1, YTHDC2, and ZC3H13 expression levels were significantly greater in tumor tissues (*p* < 0.05, **Figures 2B, C**). The findings suggested that m6A regulators may play a role in the biological development of cervical cancer.

### Correlation and functional enrichment between m6A RNA methylation regulators

**Figure 3:**
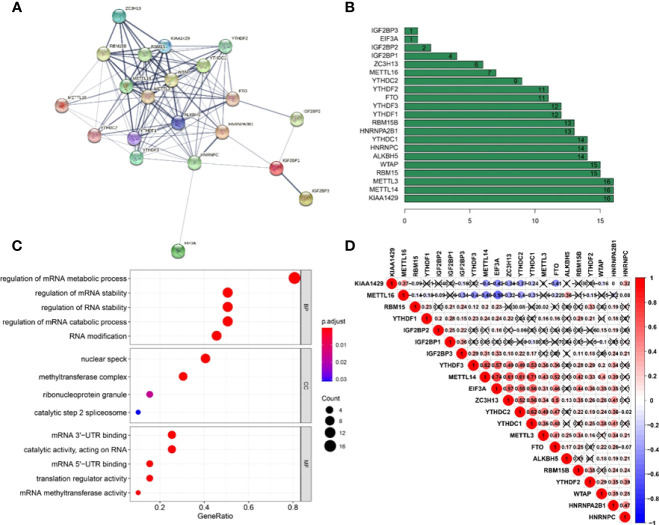
Interaction and correlation between m6A RNA methylation regulators in cervical cancer. **(A)** A PPI network was constructed to evaluate the interaction between m6A RNA methylation regulators. **(B)** Histogram of key M6A regulators. **(C)** Functional annotation of 21 m6A methylation regulators. **(D)** The correlations among m6A RNA methylation regulators were analyzed by Pearson correlation.

A PPI network was created using the STRING database to better understand the interactions between the 21 m6A methylation regulators. We discovered that the PPI network included 21 nodes and 111 edges after eliminating all separated elements with no connection (**Figure 3A**). The hub genes in the interaction network were found to be KIAA1429, METTL14, and METTL3 (**Figure 3B**). These genes were highly enriched in mRNA metabolic process regulation, mRNA stability regulation, RNA stability regulation, mRNA catabolic process regulation, and RNA modification (**Figure 3C**). Furthermore, we discovered that the majority of m6A RNA methylation regulators were positively associated with METTL14 having the strongest connection with EIF3A (**Figure 3D**) (*r* = 0.74).

### The consensus cluster of m6A methylation regulators was significantly associated with clinical signatures of cervical cancer patients

**Figure 4:**
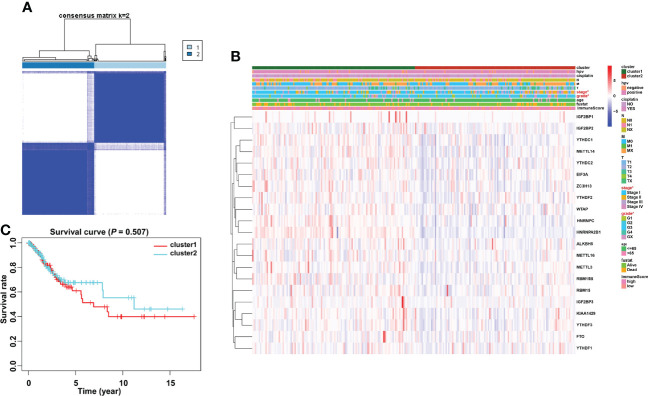
Consensus clustering identified two cervical cancer patient clusters and their relationship with clinicopathological parameters. **(A)** The cervical cancer cohort from TCGA was divided into two distinct clusters when *k* = 2. **(B)** Comparison of the relationship between the clinicopathological characteristics of two clusters. **(C)** The clinicopathological features between the two subtypes were then compared. Cluster 2 was preferentially associated with a low WHO stage and grade (*p* < 0.05).

Based on the similarity of the expression level of m6A regulators and the proportion of fuzzy clustering measures, it is established that *k* = 2 has the best clustering stability from *k* = 2 to 9 (**Supplementary Figure 2**). A total of 304 cervical cancer patients were divided into clusters 1 and 2 (N1 = 152, N2 = 152) based on the expression levels of the m6A regulators (**Figure 4A**). The results showed that m6A methylation regulators were expressed at a higher level in cluster 1 than in cluster 2 (**Figure 4B**). Furthermore, the clinical prospects of clusters 1 and 2 were compared. The WHO stage and grade of cervical cancer differed considerably between the two groups (*p* < 0.05, **Figure 4B**). However, the OS did not differ considerably across the two clusters (**Figure 4C**).

### Correction between PD-L1 and m6A methylation regulators

**Figure 5:**
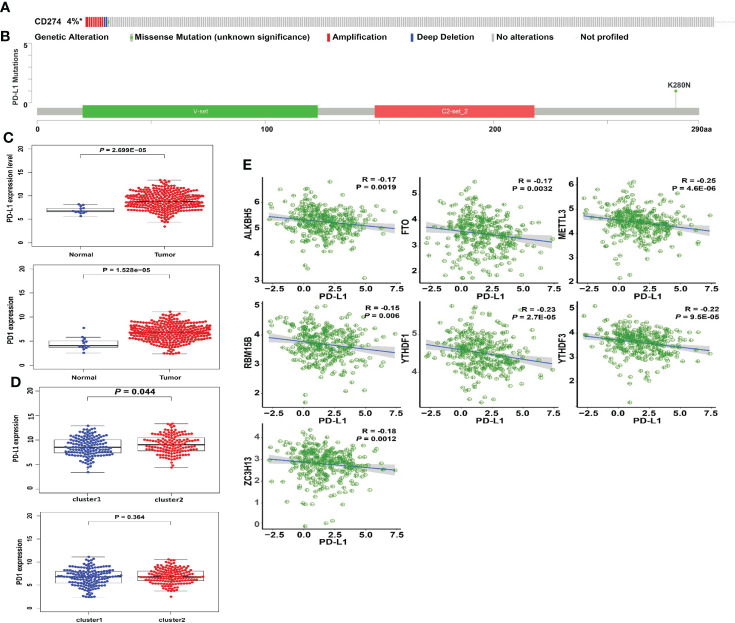
Association of PD-L1 with m6A RNA methylation and the landscape of immune cell infiltration in cervical cancer. **(A)** OncoPrint of PD-L1 alterations in the TCGA-CESC cohort identified by cBioPortal. **(B)** Lollipop of PD-L1 alterations in the TCGA-CESC cohort identified. **(C)** PD-L1 expression was significantly higher in cervical cancer than that in controls. **(D)** The expression level of PD-L1 in cluster 1/2 subtypes. **(E)** The correlation of PD-L1 with m6A methylation regulators in cervical cancer.

The types and frequency of PD-L1 mutations in cervical cancer were investigated using the cBioPortal platform. PD-L1 is mutated in PD-L1 is mutated in 4% of cervical cancer patients, including missense mutations, amplifications, and deep deletions (**Figure 5A**). Amplifications account for the vast majority of PD-L1 changes in cervical cancer. The sites of PD-L1 mutations in cervical cancer patients were visualized using a lollipop diagram (**Figure 5B**). We evaluated the difference in PD-L1 expression between tumor samples and controls, clusters 1 and 2, and high- and low-risk groups to determine the link between PD-L1 and m6A regulators. In cervical cancer samples, PD-L1 expression was much higher than in normal surrounding tissues (**Figure 5C**, *p* < 0.001). The expression of PD-L1 in clusters 1 and 2 differs statistically significantly (**Figure 5D**). Furthermore, ALKBH5, FTO, METTL3, RBM15B, YTHDF1, YTHDF3, and ZC3H13 were all inversely linked to PD-L1 (*p* < 0.01, **Figure 5E**).

### Association of distinct immune cell infiltration with m6A methylation regulators

**Figure 6:**
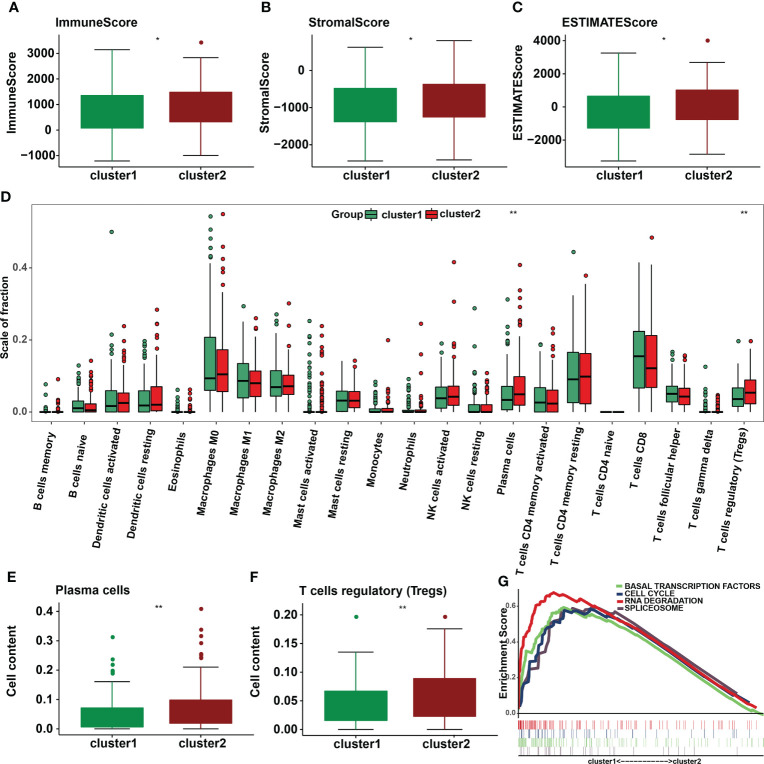
Differences in the level of immune cell infiltration between the two clusters in cervical cancer and the biological pathways involved. **(A)** Immunescore, **(B)** stromalscore, and **(C)** estimatescore in the cluster 1/2 subtypes. **(D)** The infiltrating levels of 22 immune cell types in cluster 1/2 in cervical cancer. **(E)** The infiltrating levels of the plasma cells and **(F)** regulatory T cells in two clusters. *p < 0.05; **p < 0.01. **(G)** GSEA shows signaling pathways involved in cluster 1.

Immune cells and stromal cells are two important non-tumor components of the TIME (18). To investigate the relationship between m6A regulators and TIME in cervical cancer, we scored immune cells (**Figure 6A**) and stromal cells (**Figure 6B**) in each sample and summed the two scores to get the total estimatescore (**Figure 6C**). A lower tumor purity was associated with higher total scores. According to our findings, immunescores, stromalscores, and estimatescores were all higher in cluster 2 (*p* < 0.05). The infiltration levels of 22 immune cell subtypes were then compared between clusters 1 and 2 (**Figure 6D**). Cluster 2 had greater amounts of plasma cell (*p* = 0.0028) and Treg (*p* = 0.0051) infiltration (**Figures 6E, F**). GSEA was utilized to determine the underlying regulatory mechanisms that result in temporal differences between clusters 1 and 2. Lastly, the findings revealed that cluster 1 was associated with basal transcription factors, the cell cycle, RNA degradation, and the spliceosome (**Figure 6G**).

### Accurate prognostic prediction of signatures for m6A methylation regulators

**Figure 7:**
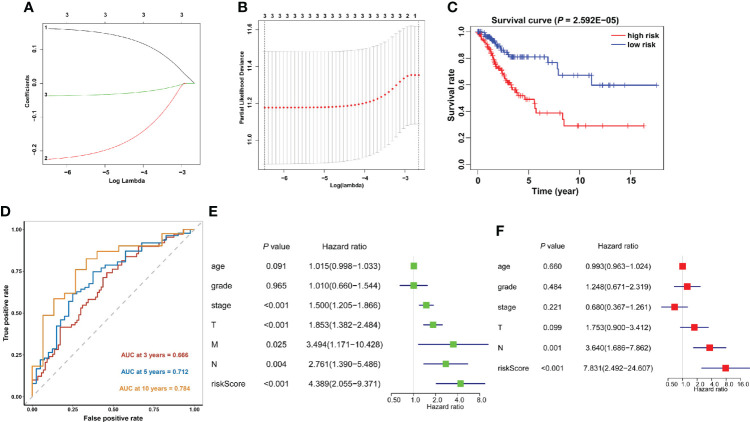
Construction of the prognostic signature based on the TCGA cervical cancer cohort. **(A, B)** The prognostic signature constructed by the minimum criterion of the LASSO Cox regression algorithm. **(C)** The Kaplan–Meier curve shows that the riskscore based on the prognostic signature of m6A RNA methylation is significantly correlated with OS in cervical cancer patients. **(D)** Time-dependent ROC curves were applied to assess the predictive efficiency of the signature in TCGA. **(E)** Univariate and **(F)** multivariate Cox regression analysis of the riskscores in TCGA.

The predictive efficacy of these 21 m6A methylation regulators in cervical cancer was next investigated using univariate Cox regression analysis. Then, three m6A regulators were recognized: METTL16, YTHDF1, and ZC3H13 (**Supplementary Table 2**). The LASSO technique was used to calculate the coefficient of each prognostic gene (**Figures 7A, B**). Three m6A regulators (METTL16, YTHDF1, and ZC3H13) were chosen as the minimum standard for constructing a predictive signature, and the riskscore of each cervical cancer patient was determined. Riskscore = (0.0369 * YTHDF1 expression) + (0.2248 * METTL16 expression) + (0.1619 * ZC3H13 expression). The patients were then separated into high- and low-risk groups based on their median riskscore. Furthermore, Kaplan–Meier curve analysis revealed that the high-risk group had a worse prognosis than the low-risk group (**Figure 7C**). Following that, a time-dependent ROC curve was built to assess the specificity and sensitivity of prognostic signals related with m6A methylation regulators. The AUC of three risk signatures was 0.666, 0.712, and 0.784 at 3, 5, and 10 years, respectively (**Figure 7D**). Three risk signatures demonstrated a good predictive power in the prognosis of cervical cancer. Furthermore, we used the TCGA-CESC cohort to do univariate and multivariate Cox regression analysis to see if the riskscore based on prognostic markers is an independent prognostic indicator for cervical cancer patients. Univariate analysis revealed that the riskscore (*p* = 0.002, HR = 5.203), tumor stage (*p* = 0.001, HR = 1.515), T stage (*p* = 0.006, HR = 1.436), M stage (*p* = 0.005, HR = 3.175), and N stage (*p* = 0.004, HR = 2.627) were significantly correlated with OS, and subsequent multivariate Cox regression analyses indicated that the riskscore (*p* = 0.001, HR = 7.830) and N stage (*p* = 0.001, HR = 3.640) (**Figures 7E, F**) were independent prognostic factors for cervical cancer.

### Riskscores are related to clinical features in cervical cancer

**Figure 8:**
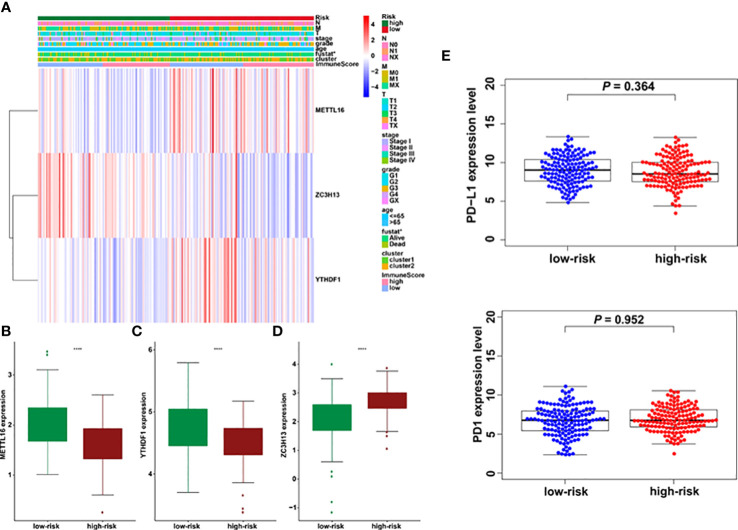
Prognostic riskscores correlated with stage, immunescore, and clinicopathological parameters in cervical cancer. **(A)** Heatmap and clinicopathologic parameters of high- and low-risk groups. **(B–D)** The differences of METTL16, YTHDF1, and ZC3H13 expression in low- and high-risk groups. **(E)** The relationship between riskscore and PD-L1 expression *p < 0.05; ****p < 0.0001.

**Figure 9:**
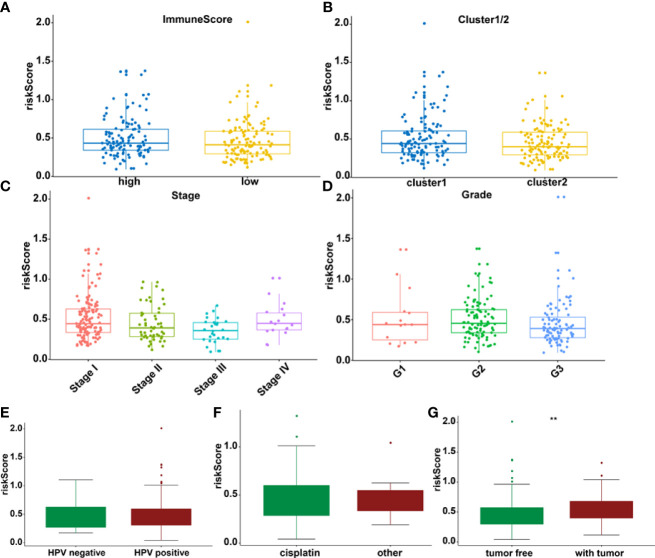
The relationship between riskscore and immunescore **(A)**, cluster 1/2 **(B)**, TNM staging **(C)**, grade **(D)**, HPV **(E)**, cisplatin **(F)**, and tumor stage **(G)** **p < 0.01.

The association between riskscore and clinical features as well as cluster subgroups was investigated further in the cervical cancer study (**Figure 8A**). METTL16 (*p* = 1.1E-08) was found to be more abundant in the high-risk group (**Figure 8B**). YTHDF1 (*p* = 3.4E-10) and ZC3H13 (*p* = 2.4E-15) were considerably lower expressed in the high-risk group (**Figures 8C, D**). Furthermore, neither PD-L1 nor the riskscore were shown to have statistical significance (**Figure 8E**). In terms of cervical cancer living stage, there was a substantial difference between the high-risk and low-risk groups (**Figure 8A**). The connection between riskscore and immunescore, cluster, TNM stage, grade, HPV stage, cisplatin use, and tumor size were also investigated (**Figures 9A–G**). Even though the differences in immunescores and riskscores were not significant, the high immunescore group had a higher median riskscore than the low immunological score group (**Figure 9A**). Furthermore, no statistically significant differences were detected in cluster 1/2 (**Figure 9B**), TNM stage (**Figure 9C**), grade (**Figure 9D**), HPV stage (**Figure 9E**), cisplatin use (**Figure 9F**). The data demonstrated that after treatment, cervical cancer patients with tumors had a considerably higher riskscore than those without tumors (*p* = 0.002, **Figure 9G**). The association between TMB and m6A regulators was also investigated in our study; however, no statistically significant variations in immunescore, PD-L1 expression, riskscore, or clustering were found between the high TMB and low TMB groups (**Supplementary Figure 2**). These data suggest that the riskscore of cervical cancer patients may have a significant impact on clinical outcomes.

### The relationship between m6A regulator signature genetic alterations and immune cell infiltration

**Figure 10:**
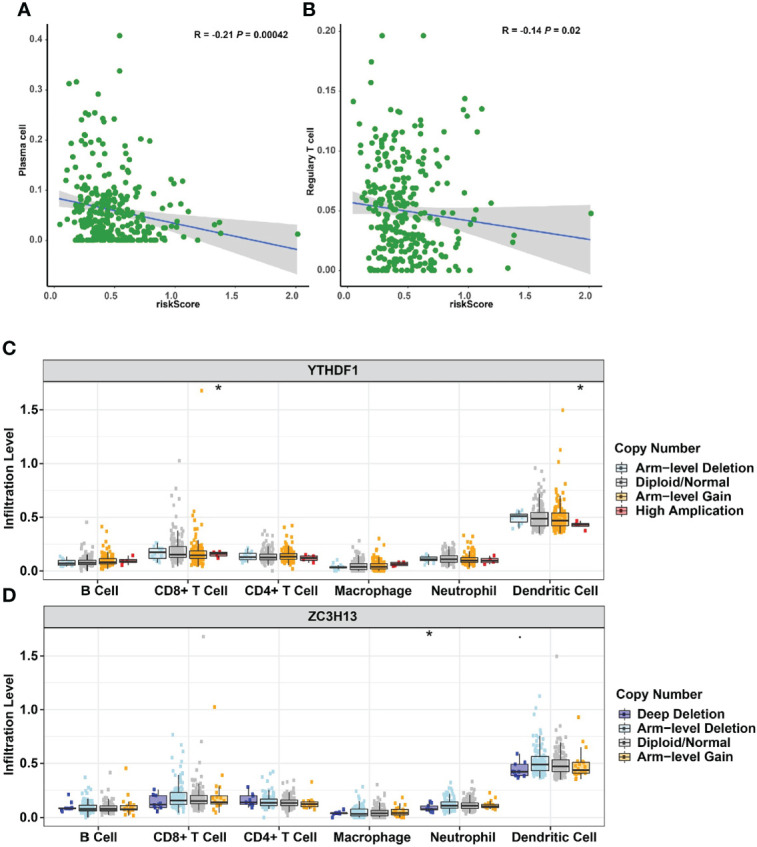
Relationships between the riskscore and infiltration abundances of nine immune cell types. **(A)** Plasma cells. **(B)** Regulatory T cells. Effect of the genetic alterations of m6A regulator-relevant signature on the immune cell infiltration. **(C)** YTHDF1. **(D)** ZC3H13. *p < 0.05.

The impact of three m6A methylation regulators on the cervical cancer TIME was evaluated by examining the link between the riskscores of 22 immune cell types and the extent of infiltration. The riskscore was found to be negatively correlated with plasma cell infiltration (*p* = 4.2E-4, **Figure 10A**) and Treg infiltration (*p* = 0.02, **Figure 10B**). The cervical cancer TIME was associated with risk signatures based on m6A regulators. Furthermore, the influence of somatic CNA based on m6A modulator signal on immune cell infiltration was investigated to preliminarily elucidate the potential mechanism of riskscore and diverse immune cell infiltration. The infiltration of CD8 + T cells, dendritic cells, and neutrophils in cervical cancer was dramatically affected by the detected CNAs of m6A regulator signatures, including arm-level deletion, diploid/normal, arm-level gain, high amplification, and deep deletion (**Figures 10C, D**). This study adds to the evidence that m6A methylation regulators play a significant role in the TIME of cervical cancer patients.

### The association between m6A and PD-L1 in cervical cancer tissues and cells

**Figure 11:**
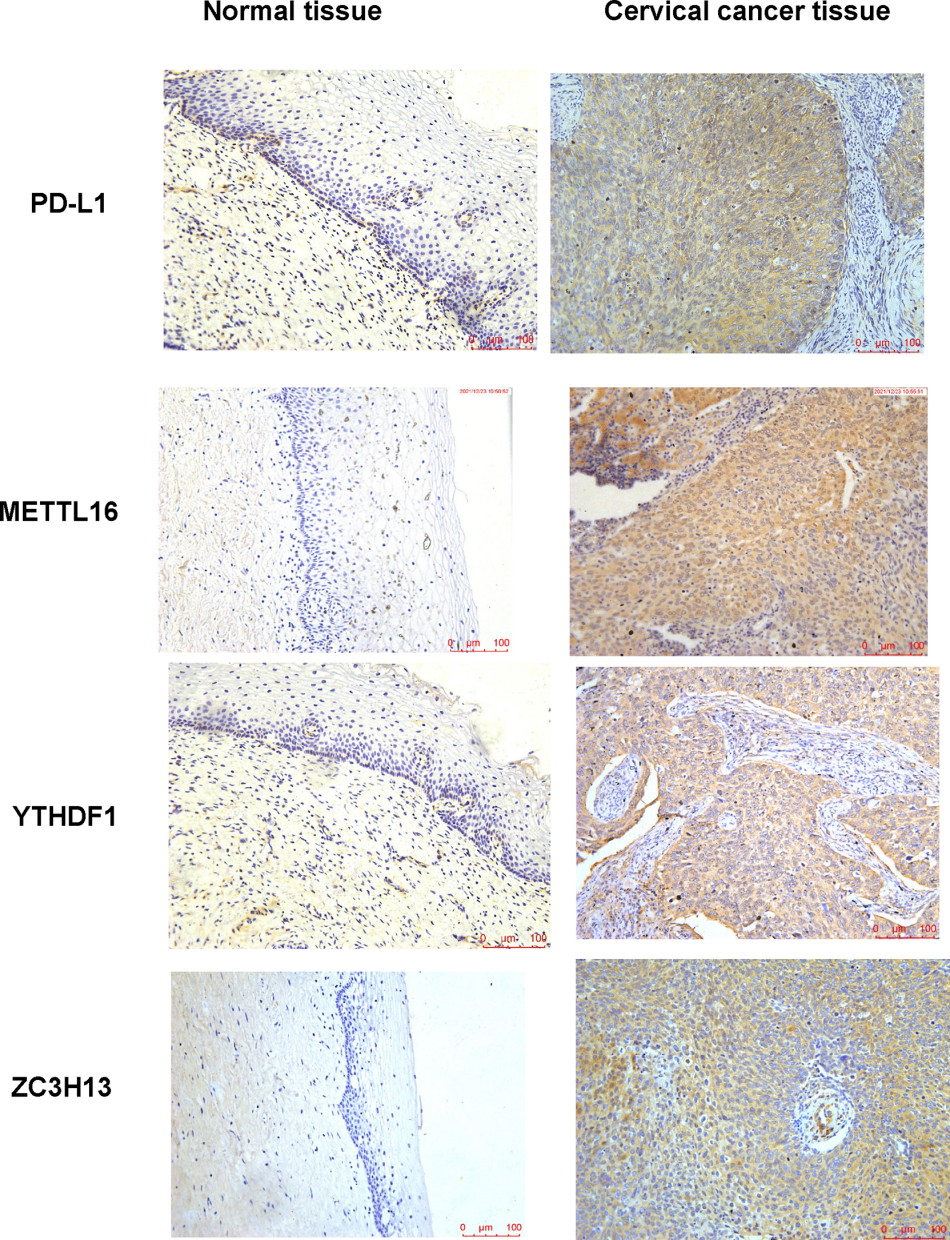
IHC was performed to measure the expression of PD-L1, METTL16, YTHDF1, and ZC3H13 in cervical cancer tissues and surrounding normal tissues.

**Figure 12:**
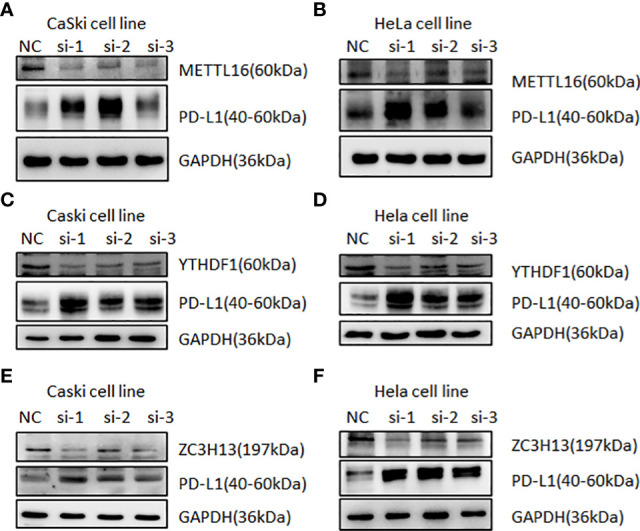
Western blotting was used to detect the relationship between m6A regulators and PD-L1 in cervical cancer. Immunoblot of METTL16, YTHDF1, ZC3H13, and PD-L1 in CaSki cells and HeLa cells after depletion of METTL16 **(A, B)**, YTHDF1 **(C, D)**, and ZC3H13 **(E, F)**. GAPDH was used as a loading control.

In human cervical cancer tissues, the expression of PD-L1, METTL16, ZC3H13, and YTHDF1 was highly expressed in cervical cancer patients compared with surrounding normal tissues (**Figure 11**). Moreover, downregulation of METTL16, YTHDF1, or ZC3H13 in two cervical cancer cell lines elevated the expression of PD-L1 (**Figure 12**). This study indicated that METTL16, YTHDF1, and ZC3H13 could regulate the expression of PD-L1 in cervical cancer.

## Discussion

The main treatments for cervical cancer are surgery in combination with radiotherapy, chemotherapy, and targeted therapy. The survival rate for recurring is still a big concern (21). Although relevant immunotherapies for cervical cancer are still in the early stages of development, some related inhibitors have entered clinical trials and showed long-term anticancer effectiveness with manageable side effects, indicating that the immunological microenvironment of cervical cancer is worth investigating further (22, 23). Simultaneously, m6A methylation, the most prevalent type of mRNA alteration, has been shown to promote or repress cancer in a variety of tumor types (24, 25), but there have been very few investigations in cervical cancer. Therefore, there is a need to further explore the role of m6A methylation in cervical cancer and the impact on the TIME infiltration of cervical cancer. Furthermore, the impact of m6A methylation on cervical cancer TIME is still unknown. Therefore, the expression patterns, prognostic values, and impacts of m6A RNA methylation regulators on TIME in cervical cancer were investigated.

With the exception of FTO, the expression levels of m6A regulators in cervical cancer were much greater than in normal tissues. METTL3, METTL14, and WTAP methylation levels in cervical cancer were reported to be considerably greater than in surrounding normal tissues in a prior investigation (17, 26). Furthermore, high levels of METTL3 and YTHDF1 expression in cervical cancer patients were linked to a poor prognosis (16, 17). According to a recent study, KCNMB2-AS1 and IGF2BP3 established a positive regulatory circuit that increased KCNMB2-AS1’s tumorigenic activity in cervical cancer (27). Moreover, one study reported that knocking down IGF2BPs greatly reduced MYC expression and hindered cancer cell proliferation, colony forming ability, and cell migration/invasion, mimicking the effect of MYC silencing (28). A PPI network containing 21 m6A RNA methylation regulators was created in STRING, and the biological roles of the regulators were analyzed by using GO functional annotation.

Consensus clustering of 21 m6A methylation regulators was then used to identify two molecular subtypes (clusters 1/2). Cluster 2 was confirmed to be associated with low tumor stage and grade. In terms of prognosis, no significant difference was found in OS between two subtypes. PD-L1 was overexpressed in cervical tumor tissues. Furthermore, PD-L1 was found to be associated with ALKBH5, FTO, METTL3, RBM15B, YTHDF1, YTHDF3, and ZC3H13. Further research is needed to be study whether these regulatory parameters predict the success of immunotherapy in patients with cervical cancer. We also investigated immune cell infiltration in cervical cancer patients. Cluster 2 had a higher amount of plasma cell and Treg infiltration than cluster 1. Tregs are critical for maintaining immunological self-tolerance to self-antigens and preventing immune diseases, and cervical cancer patients with increased Treg infiltration have a worse prognosis. Cluster 2 also has much higher immunological and stromalscores by using the ESTIMATE technique, indicating a significant difference in cervical cancer patients’ TIME. Furthermore, GSEA revealed that basal transcription factors, cell cycle, RNA degradation, and the spliceosome were among the signature pathways engaged in cluster 1.

Furthermore, this study created a three-gene prognostic marker consisting of an m6A methylation regulatory factor, namely, METTL16, YTHDF1, and ZC3H13, and the calculated riskscore had a good predictive effect on cervical cancer patients. Riskscore and N stage were independent prognostic factors. The high riskscore of cervical cancer patients is associated with poor prognosis and is an independent prognostic factor. Among these risk factors, elevated YTHDF1 expression was linked to a poor prognosis in cervical cancer patients, and it was thought to be an oncogene in the disease (17, 29). YTHDF1 aggravated the carcinogenesis of cervical cancer *via* m6A-induced promotion of RANBP2 (16). Another study discovered that the YTHDF1/eEF-2 complex and IGF2BP3 increase the translation elongation and mRNA stability of pyruvate dehydrogenase kinase 4 to control glycolysis in cervical cancer cells (30). Moreover, METTL16-mediated m6A methylation boosted gastric cancer cell proliferation by increasing cyclin D1 expression (31). Furthermore, METTL16 was shown to be substantially expressed in gastric cancer and colorectal cancer cells, and was linked to a poor prognosis (31, 32). In contrast, increased METTL16 expression predicts a longer survival in individuals with liver cancer (33). Notably, accumulating evidence showed that METTL16 has been associated with a bad prognosis and has been found to be highly expressed in gastric cancer and colorectal cancer cells (31, 32). However, one study suggested that greater METTL16 expression predicted a longer survival in people with liver cancer (33). The fact that the same gene encoding the methyltransferase has different functions in various malignancies could explain the difference in METTL16’s prognostic efficacy between gastric cancer and liver cancer (13). ZC3H13 expression was shown to be drastically reduced in endometrial carcinoma tissues in a prior study, and it was found to inhibit endometrial carcinoma cell lines from increasing proliferation and invasion (34). ZC3H13 enhanced stemness and chemoresistance *via* modulation of CENPK mRNA in cervical cancer cells (35). However, the relevance of METTL16 and ZC3H13 in cervical cancer is still not fully known.

Recent studies have also focused on immune-related prognostic features associated with cancer immune invasion (36). For example, a growing body of literature strongly suggested that YTHDF1 may regulate the immune microenvironment of breast cancer, influencing tumor growth as well as immunotherapy efficacy (36). According to a recent study, YTHDF1 and YTHDF2 induced inflammation in the TIME in non-small-cell lung cancer (37). Another study found that YTHDF1 was involved in the regulation of long-term neoantigen-specific immunity, and YTHDF1-deficient mice had an increased antigen-specific CD8+ T-cell antitumor response in colon cancer (38). The research on the other two risk signatures, as well as immune cell infiltration, is still in the early stages. In terms of additional m6A regulators, one study observed that in mice tumors missing YTHDF1, the degree of CD8+ T and NK cell infiltration was increased, enhancing *in vivo* tumor antigen cross-expression and CD8+ T-cell cross-priming (38). The loss of METTL3 or METTL14 causes T-cell proliferation and differentiation to be disrupted, lowering the sensitivity of interleukin 7 (IL-7) *in vivo* (39). Our findings also revealed that CNAs of m6A methylation regulators, such as arm-level deletion, diploid/normal, arm-level gain, high amplification, and deep deletion, had a substantial impact on CD8+ T cells, dendritic cells, and neutrophil infiltration in cervical cancer. TIME is believed to be regulated by the m6A methylation regulator in cervical cancer patients.

There are various limitations to this study. Firstly, the small sample sizes may have an impact on the results. Future studies must increase the sample size, sequencing data, and clinical information of cervical cancer patients. Furthermore, our findings are based on bioinformatic analysis of datasets containing genetic and other molecular information from patient tissues, which will need to be validated using cell lines, animal models, and clinical trials. In conclusion, the research looked at the expression of m6A RNA regulators in cervical cancer, their relationship with PD-L1, and putative regulation mechanisms. The difference in the degree of immune cell infiltration in the TIME was assessed using consensus clustering of m6A regulators. The m6A regulators may boost immunotherapy response in cervical cancer patients by modulating TIME and PD-L1 expression. More importantly, we created a prognosis marker incorporating three m6A RNA methylation genes and identified the riskscore as an independent prognostic factor in the cervical cancer cohort, indicating that prognostic markers are a viable tool for predicting survival outcomes in cervical cancer patients.

## Data availability statement

The original contributions presented in the study are included in the article/**Supplementary Material**. Further inquiries can be directed to the corresponding authors.

## Ethics statement

The studies involving human participants were reviewed and approved by the Second Affiliated Hospital of Wenzhou Medical University. The patients/participants provided their written informed consent to participate in this study.

## Author contributions

Conceptualization, HJ and XZ; methodology, HJ; formal analysis, HJ, JZ, HL, and KL; writing, HJ, ZW, and XZ; visualization and supervision, XZ. All authors have read and agreed to the published version of the manuscript.

## Funding

This work was supported by a grant from the National Natural Science Foundation of China (No. 82172088).

## Acknowledgments

The graphical abstract was created with BioRender.com. The authors would like to thank the TCGA, GTEx, cBioPortal, and TIMER databases for open access.

## Conflict of interest

The authors declare that the research was conducted in the absence of any commercial or financial relationships that could be construed as a potential conflict of interest.

## Publisher’s note

All claims expressed in this article are solely those of the authors and do not necessarily represent those of their affiliated organizations, or those of the publisher, the editors and the reviewers. Any product that may be evaluated in this article, or claim that may be made by its manufacturer, is not guaranteed or endorsed by the publisher.
