# Supplementary material for: Comprehensive characterization of tumor microenvironment and m6A RNA methylation regulators and its effects on PD-L1 and immune infiltrates in cervical cancer
Source: Front Immunol. 2022 Aug 26;13:976107. doi: 10.3389/fimmu.2022.976107 (PMC9458859; doi:10.3389/fimmu.2022.976107)
Supplement: Supplementary file 1 [file DataSheet_1.pdf]

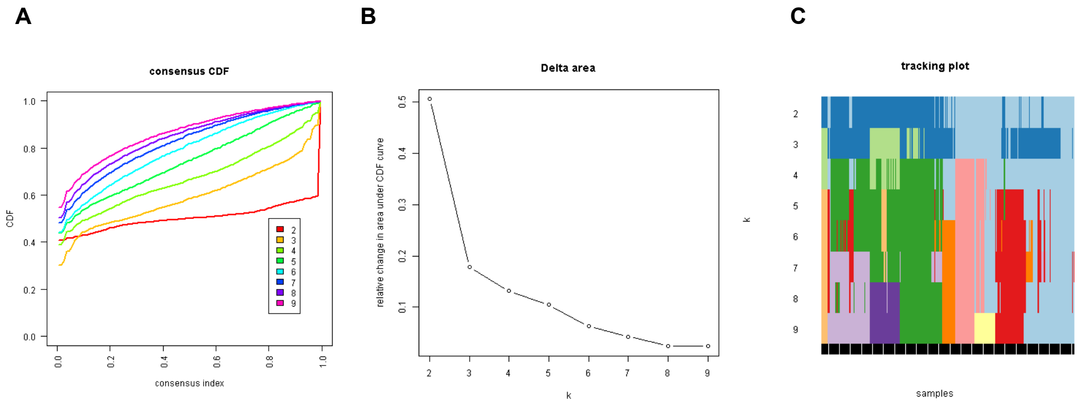

Supplementary figure 1: Consensus clustering identified two cervical cancer patient clusters. (A) Consensus clustering cumulative distribution function (CDF) for k=2 to 9; (B) relative change in area under CDF curve for k=2 to 9; (C) distribution of each sample when k ranges from 2 to 9.

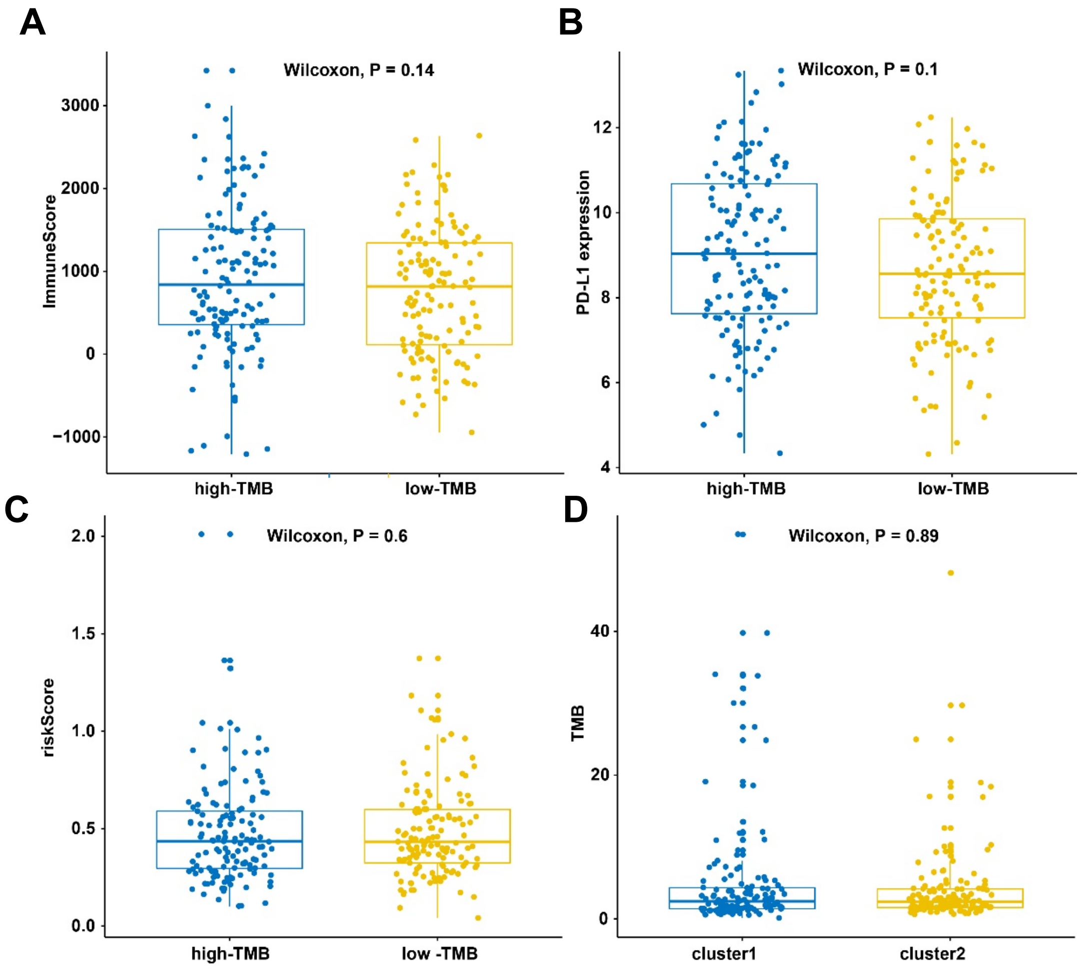

Supplementary figure 2: The relationship between TMB and immunescore (A), PD-L1 expression (B), riskscore (C) and cluster 1/2 (D).
