# Supplementary material for: Comprehensive characterization of tumor microenvironment and m6A RNA methylation regulators and its effects on PD-L1 and immune infiltrates in cervical cancer
Source: Front Immunol. 2022 Aug 26;13:976107. doi: 10.3389/fimmu.2022.976107 (PMC9458859; doi:10.3389/fimmu.2022.976107)
Supplement: Supplementary file 2 [file Table_1.docx]

Supplementary Table 1: The siRNA sequences for transfection.

| siRNA | Sequence (5'-3') |
| --- | --- |
| siMETTL16-1 | CCUUGAGACUCAACUAUAUTT |
| siMETTL16-2 | CAUAGUCGUUGUCACGACATT |
| siMETTL16-3 | CCAUGACAGUCUACAACUUTT |
| siYTHDF1-1 | GUUCGUUACAUCAGAAGGAUA |
| siYTHDF1-2 | CGGUGGGACAAAUGUGAACAU |
| siYTHDF1-3 | GGCUGGAGAAUAACGACAATT |
| siZC3H13-1 | CCUCACAAUCAGGAUCAUCUA |
| siZC3H13-2 | GGAUCGAGAUAGAUUGCGATT |
| siZC3H13-3 | CCAAGUUAGAUGAUGCACAUU |
| siRNA-NC | UUCUCCGAACGUGUCACGU |
