# Supplementary material for: Comprehensive characterization of tumor microenvironment and m6A RNA methylation regulators and its effects on PD-L1 and immune infiltrates in cervical cancer
Source: Front Immunol. 2022 Aug 26;13:976107. doi: 10.3389/fimmu.2022.976107 (PMC9458859; doi:10.3389/fimmu.2022.976107)
Supplement: Supplementary file 3 [file Table_2.docx]

Supplementary table 2. Univariate analysis of 21 m6A RNA methylation regulators.

| Gene | HR | HR.95L | HR.95H | P value |
| --- | --- | --- | --- | --- |
| ALKBH5 | 0.991614 | 0.969317 | 1.014424 | 0.467976 |
| EIF3A | 1.011855 | 0.994348 | 1.02967 | 0.185686 |
| FTO | 0.955101 | 0.781963 | 1.166574 | 0.652593 |
| HNRNPA2B1 | 1.004693 | 0.996432 | 1.013022 | 0.266365 |
| HNRNPC | 1.009034 | 0.993027 | 1.025299 | 0.27031 |
| IGF2BP1 | 1.034785 | 0.963546 | 1.11129 | 0.347442 |
| IGF2BP2 | 1.009034 | 0.991248 | 1.027139 | 0.321618 |
| IGF2BP3 | 0.955219 | 0.826905 | 1.103444 | 0.533618 |
| KIAA1429 | 1.07539 | 0.962776 | 1.201176 | 0.197805 |
| METTL14 | 1.014175 | 0.794624 | 1.294386 | 0.909969 |
| METTL16 | 0.830083 | 0.67644 | 1.018624 | 0.074543 |
| METTL3 | 1.019496 | 0.931189 | 1.116176 | 0.676171 |
| RBM15 | 0.978029 | 0.811781 | 1.178322 | 0.815205 |
| RBM15B | 1.00139 | 0.944782 | 1.06139 | 0.96268 |
| WTAP | 1.016914 | 0.976338 | 1.059177 | 0.419466 |
| YTHDC1 | 0.977068 | 0.924564 | 1.032553 | 0.41038 |
| YTHDC2 | 0.952599 | 0.803733 | 1.129038 | 0.575405 |
| YTHDF1 | 0.970459 | 0.938534 | 1.003471 | 0.078929 |
| YTHDF2 | 1.006383 | 0.968585 | 1.045656 | 0.7446 |
| YTHDF3 | 1.044065 | 0.976625 | 1.116163 | 0.205616 |
| ZC3H13 | 1.117795 | 1.024811 | 1.219216 | 0.011969 |
